# An observation on the severity of periodontal disease in past cigarette smokers suffering from rheumatoid arthritis- evidence for a long-term effect of cigarette smoke exposure?

**DOI:** 10.1186/s12903-018-0531-5

**Published:** 2018-05-10

**Authors:** Márk Antal, Emese Battancs, Márta Bocskai, Gábor Braunitzer, László Kovács

**Affiliations:** 1 Faculty of Dentistry, Department of Aesthetic and Operative Dentistry, University of Szeged, 6720 Tisza Lajos körút, Szeged, 64 Hungary; 2 Faculty of Medicine, Department of Rheumatology and Immunology, University of Szeged, 6725 Kálvária sugárút, Szeged, 57 Hungary; 3 Laboratory for Perception & Cognition and Clinical Neuroscience, Nyírő Gyula Hospital, 1135 Lehel utca, Budapest, 59 Hungary

**Keywords:** Rheumatoid arthritis, Periodontal disease, Chronic inflammation, Late sequelae, Tobacco smoking

## Abstract

**Background:**

Rheumatoid arthritis (RA) and cigarette smoking are both risk factors for periodontal disease (PD). Previous research suggests that systemic inflammatory conditions and cigarette smoking may act in synergy, and their co-occurrence leads to a much higher risk of developing severe stage PD than what the combination of their individual risks would suggest. We originally sought to test this in the case of RA, but it turned out that the majority of our patients were former smokers, who smoked for prolonged periods in the past. For that reason, we decided to shift our focus toward the possible effects of past chronic cigarette smoke exposure.

**Methods:**

The data of 73 RA patients and 77 healthy controls were analyzed. The participants received a full-mouth periodontal examination to determine their periodontal status. Rheumatological indices and data on past tobacco use were also recorded. Both the patient and the control groups were divided into former smoker and non-smoker subgroups for the analyses. Non-smoker controls were used as the reference group.

**Results:**

In the control group, smoking in history increased the odds of developing both the moderate and the severe stages of PD, but the change was not statistically significant. RA significantly, increased the odds of developing both stages in itself, but the highest odds were seen in the former smoker RA group.

**Conclusion:**

Based on this surprising observation of ours, we hypothesize that chronic cigarette smoke might bring about permanent changes in the periodontal tissues, leading to their hypersensitivity to inflammatory challenges.

## Background

The connection between periodontal disease (PD) and various systemic conditions of an autoimmune/dysimmune background is well documented [1–5]. Rheumatoid arthritis (RA) is an immune mediated disease with a particularly well established link to PD [6–9]. While the exact immunological mechanisms have not been clarified, there is evidence to suggest that the presence of citrullinated proteins (and antibodies against them) is the link [10–12]. In this respect, the role of the periodontal pathogen, *Porphyromonas gingivalis* is emphasized, the only periodontal pathogen that expresses the citrullinating enzyme peptidyl-arginine deiminase (PPAD) [9, 13, 14]. Compared to the general population, subjects with PD are at an increased risk of developing RA, and vice versa [9, 15], which suggests that once they are established, they mutually aggravate each other.

Cigarette smoking is a known risk factor for both PD [16–18] and RA [19–21]. Cigarette smoking promotes oral bacterial colonization [22] and smoking itself has been shown to promote citrullination [23], evidenced by the fact that an association was found between tobacco exposure and anti-cyclic citrullinated peptide (anti-CCP) titers in RA patients [24]. It follows that cigarette smoking may be an additional aggravating factor in both PD and RA, especially when the two conditions are comorbid.

In 2014, we published a study about the effects of cigarette smoking on the severity of PD in psoriasis [25]. In that study, we found that while both psoriasis and smoking significantly elevated the odds that the individual will develop advanced PD, when both risk factors were present, the odds multiplied, well beyond the combined odds. We concluded that cigarette smoking probably acted as a permissive factor, and we articulated a hypothesis about the possible role of toll-like receptor 4 (TLR-4). The hypothesis was that smoking exerts this effect through the upregulation of TLR-4.

In their review [26], Baka and co-workers point out that as cigarette smoking promotes bacterial colonization, it may well be that smokers are exposed to an increased burden of *P. gingivalis*, whereby they are constantly exposed to a potent antigen.

Based on these premises, we wished to find out about if smoking acts as a booster of periodontal deterioration also in the context of RA. This is an important question with practical bearings. For instance, severe PD was reported to hamper the efficacy of anti-TNF (tumor necrosis factor) therapy [27].

To answer the question, we collected data on 82 RA patients and 100 controls who met all the inclusion and exclusion criteria and also gave their informed consent. At this point, though, we were faced with a difficulty: most of our patients turned out to be non-smokers (NS). Only eight of them smoked at the time of the study, and the rest had either quit or never ever smoked in their lives. The majority of our patient sample, therefore, consisted of non-smokers and former smokers (FS) who had quit smoking long before.

While we have a wealth of information about the immunological consequences of current smoking [28–31], we know almost nothing about the permanent immunological changes that chronic exposure to cigarette smoke may bring about - and that may remain even if the smoker quits. Considering that the majority of our formerly smoking patients had smoked for at least a decade before they quit, and that chronic exposure to cigarette smoke was proven to induce genome level changes [32–34], we decided to shift our focus and concentrate on the effects of past smoking. We hypothesized that the periodontal status of patients with no smoking history would be significantly poorer than that of healthy controls (the effect of RA), and we wished to know if past smoking would be associated with poorer periodontal status to any extent. We were also interested in the relationship between the rheumatological factors and the periodontal status.

## Methods

Both RA patients and healthy controls were recruited on a voluntary basis.

Patients were eligible for the study if they met the 2010 European League against Rheumatism and American College of Rheumatology (EULAR/ACR) criteria for rheumatoid arthritis [35]. Exclusion criteria for both groups were determined based on the literature of the subject and included obesity (body mass index - BMI≥30), excessive alcohol consumption, drug abuse, diabetes mellitus, diseases causing neutropenia and local or systemic inflammatory conditions (other than RA) [36]. Poor oral hygiene, defined as Simplified Oral Health Index (OHI-S)- > 3 [37] was also an exclusion criterion.

Required sample size was calculated with G*Power 3.1.5. (University of Kiel, Germany), a software designed especially for statistical power and sample size computation [38]. The software allows the computation of achieved statistical power (post-hoc) and required sample size (a priori). As mostly categorical variables were to be analyzed, a priori sample size estimation was performed for crosstabs/chi square/contingency tables, with the following input parameters: effect size (w): 0.3; α: 0.05;power (1-β): 0.9; df: 3. Required sample size turned out to be *n* = 158 (for four groups: RA smoker/former smoker; control smoker/former smoker).

RA Patients (*n* = 82) were recruited from among the patients of the Department of Rheumatology, University of Szeged. The control group (*n* = 100) was recruited from among people attending mandatory lung screening in the same city and the same period. After removing the actual smokers from the sample, we were left with 150 participants (73 patients and 77 controls), yielding a statistical power of 0.88.

The study was approved by the Institutional and Regional Ethics Committee for Medical Biological Research at the University of Szeged (approval No.144/2014), and the study design conformed to the Declaration of Helsinki in all respects. Written informed consent was obtained from all participants.

Demographic and tobacco use data were collected by a questionnaire. Participants were divided into FS and non-smoker groups, based on their self-reported tobacco use in the past. In both the patient and the control groups, a subject was considered a FS if they smoked for at least one year in the past as a habit and without interruption. Sixty-four percent of the FS controls and 74 % of the FS RA patients provided tobacco use information that could be used for the analyses.

The clinical disease severity of PD is still a matter of debate, and several methods are available in the literature [39]. We decided to use the staging proposed by Fernandes and colleagues [40]. The reason for using this classification was that its clinical staging matches the pathological/pathophysiological changes in PD very well [41], and that we had had previous experience with it [25]. The staging requires the following parameters to be recorded: bleeding on probing (BOP; the presence or absence of bleeding within 15 s after probing), probing depth (PRD; in millimeters), and clinical attachment level (CAL; to describe the position of the soft tissue in relation to the cemento-enamel junction). All subjects received a full-mouth examination and their periodontal status was classified into one of the four categories of the staging: healthy(0); early (1); moderate (2); severe (3). For the examination, Williams probes (Hu-Friedy Manufacturing Co., Chicago, USA) were used. Table 1 shows the categories of the applied staging and the corresponding pathological/pathophysiological status. Although there is some lack of overlap in the first stage of the PD, it may not influence any of the results as nor the initial lesion, nor the gingivitis is having real influence on the immune sytem and the gingival health. Gingivitis itself is a reversible form of the disease.

**Table 1:** The applied clinical staging and the corresponding pathological/pathophysiological changes [40, 41]

CLINICAL STAGING (Fernandes et al., 2009)	PATHOLOGY/PATHOPHYSIOLOGY (Ohlrich et al., 2009)
1.NO CLINICAL SIGNS- no clinical attachment loss (CAL) or bleeding on probing (BOP)	(NO LESION- NOT CLASSIFIED EXPLICITLY IN OHLRICH ET AL.)
(GINGIVITIS-NOT CLASSIFIED EXPLICITLY IN FERNANDES ET AL.)	1. INITIAL LESION – up to 4 days following plaque accumulation. Polymorphonuclear leukocytes (PMN), complement activation, loss of connective tissue. Mast cells release tumor necrosis factor alpha, PMNs migrate into the gingival sulcus, but as the bacteria are protected by the biofilm, abortive phagocytosis occurs. PMNs release lysosomal contents, which leads to further tissue destruction.
2.EARLY PERIODONTITIS- CAL ≥1 mm in ≥2 teeth	2. EARLY/STABLE LESION- 7-21 days after plaque accumulation, clinically evident approximately from day 12. Dominantly macrophages and lymphocytes (CD4^+^:CD8^+^ 2:1). Perivascular inflammatory infiltrate. Intercellular spaces between epithelial cells widen, bacterial products infiltrate the gingival tissues at a higher rate. Escalation of response. If plaque removed, tissue remodeling can take place.
3.MODERATE PERIODONTITIS- 3 sites with CAL ≥4 mm and at least 2 sites with probing depth (PRD) ≥3 mm	3. ESTABLISHED OR PROGRESSIVE LESION- dominantly a B cell/plasma cell response. High levels of IL-1 and IL-6: connective tissue loss, breakdown of bone.
4. SEVERE PERIODONTITIS- CAL ≥6 mm in ≥2 teeth and PRD ≥5 mm in ≥1 site	4. ADVANCED LESION- Overt loss of attachment. High levels of IL-1, TNF α and PGE_2_ stimulate fibroblasts and macrophages to produce matrix metalloproteases. The junctional epithelium progresses in apical direction (deepening periodontal pocket). Oligoclonal Th_2_ (CD4^+^) dominance.

To characterize the patient population from a rheumatological point of view, the following indices and laboratory values were recorded: IgM rheumatoid factor seropositivity and levels (RF) measured with nephelometry, anti-citrullinated peptide antibody (ACPA) seropositivity and levels with antigenic specificity to mutated citrullinated vimentin (aMCV) measured with ELISA, disease activity score (DAS28-ESR) at the latest visit and its average of the past 12 months, and the HAQ-DI disability index. Data on the conventional and biological disease modifying antirheumatic drug (DMARD) and corticosteroid therapy of the patients were also recorded. Laboratory values were determined as part of the routine examinations (i.e. not especially for this study).

We divided the subjects into four groups based on the presence/absence of RA and smoking in the past. To express the odds that a member of a given group develops a given clinical degree of periodontal disease, multinomial logistic regression analysis was conducted and the odds ratios were calculated. In the multinomial model, disease severity (healthy, early, moderate, severe) was defined as the outcome variable, group was the factor, and age and sex were covariates. Within-group analyses (Mann-Whitney U tests) were also performed in the patient group, according to the smoking status, to see if past smoking had any effect on the rheumatological indices. For the analyses, SPSS 21.0 (IBM, USA) was used.

## Results

The demographic and tobacco use characteristics of the four studied groups are given in Table 2. It can be seen that the vast majority of the participants were females. This is because RA affects predominantly women and the control group was selected to match the patient group age- and gender-wise as closely as possible.

**Table 2:** Demographic and tobacco use characteristics of the studied groups

Group	Sex ratio F(%):M(%)	Smoke-free for (mean years, SD)	Smoked for (mean years, SD)	Cigarettes smoked per day (rounded average, SD)	Age in years (mean, SD)
CNS (*n* = 55)	44(80):11(20)	NA	NA	NA	55.7 (13.3)
CS (*n* = 22)	12(54):10 (45)	11.3(12.5)	13.8(10.2)	11 (7.9)	58.1 (13.5)
PNS (*n* = 42)	33(79): 9 (21)	NA	NA	NA	56.5 (12.7)
PS (*n* = 31)	24(77):7 (23)	16.4(12.2)	18.3(11.4)	14 (10.2)	59.3 (13.6)

*CNS* control, never smoked, *CS* control, used to smoke, *PNS* patient, never smoked, *PS* patient, used to smoke

The within-group comparisons in the patient group did not indicate significant difference in any of the rheumatological indices between FS and non-smokers (data not shown). To test the effect of RF/aMCV positivity on periodontal status, a separate multinomial regression analysis was conducted. While no statistically significant effects were found, seropositivity for RF increased the odds of the moderate stage to 1.65, and that of the severe stage to 2.51. The rheumatological indices of the patient group are summarized in Table 3.

**Table 3:** A brief rheumatological characterization of the patient population by smoking in patient history

Rheumatoid factor positivity [> 30 U/ml] n (%)	FS	20 (64.5)
	NS	24 (57.1)
Anti-citrullinated peptide antibody positivity [> 20 U/ml] n(%)	FS	20 (64.5)
	NS	25 (59.5)
Patients on conventional DMARD therapy n (%)	FS	25 (80.6)
	NS	36 (85.7)
Patients on biological DMARD therapy (%)	FS	17 (54.8)
	NS	18 (42.8)
DAS28 at visit mean (SD; range)	FS	(*n = 21*) 3.10 (1.42; 0.97–6.52)
	NS	(*n = 35*) 2.94 (1.30; 0.79–6.33)
Average DAS28 in the previous 12 months mean (SD; range)	FS	(*n = 10*) 3.20 (1.68; 1.38–5.22)
	NS	(*n = 8*) 2.41 (1.30; 1.20–5.30)
HAQ mean (SD)	FS	(*n = 15*) 0.98 (0.84)
	NS	(*n = 7*) 2.21 (0.70)

*FS* former smoker, *NS* never smoked, *DMARD* disease-modifying antirheumatic drug, *DAS28* disease activity score, *HAQ* score on the health assessment questionnaire for rheumatoid arthritis. Where data from not all patients were available, the actual number of patients is given in parentheses

The periodontal status and CAL data of each group is shown in Table 4. It is noteworthy that while the majority of the cases in both control groups falls into the healthy and early stages, in the patient groups the situation is just the opposite. This tendency is the most remarkable among the patients who used to smoke. 81% of them were classified as having moderate or severe PD. Note also that in the patient groups nobody was found who could be classified as periodontally healthy.

**Table 4:** Periodontal status in the examined groups according to Fernandes et al. (38). Data are given as n (%, rounded percentages). CAL values are also shown (mm, mean ± SD). The conventions are the same as in Table 1

	RA				Control			
	n		CAL		n		CAL	
Total	73		3.55 (±1.62)		77		2.03 (±1.17)	
	PNS		PS		CNS		CS	
	n	CAL	n	CAL	n	CAL	n	CAL
Total	42	3.18 (±1.39)	31	4.06 (±1.79)	55	2.08 (±1.26)	22	1.91 (±0.89)
Healthy	0	NA	0	NA	8 (15)	0.67 (±0.30)	4 (18)	0.76 (±0.40)
Early	12 (29)	1.81 (±0.21)	6 (19)	1.89 (±0.31)	30 (55)	1.52 (±0.39)	12 (55)	1.76 (±0.37)
Modrate	22 (52)	3.07 (±0.32)	12 (39)	3.50 (±0.89)	13 (24)	3.35 (±0.21)	5 (23)	3.19 (±0.25)
Severe	8 (19)	5.52 (±1.21)	13 (42)	5.57 (±1.46)	4 (7)	4.99 (±0.14)	1 (5)	4.72 (±NA)

The results of the multinomial regression analysis are given in Table 5. The analysis indicated no significant influence of either age or sex on periodontal status. Male sex appears to be associated with an increased risk for both the moderate and the severe stages, but given the under-representation of the male sex in this study, we would not draw conclusions from this. As for the odds of developing the moderate or severe stages, these were significantly higher in both patient groups for both stages as compared to controls who never smoked. Controls who used to smoke did not have significantly higher odds to develop any of these stages than controls who never smoked, while an increment was definitely seen. The highest significant odds ratio for the severe stage (16.25) was found in the RA group of FS.

**Table 5:** Results of the multinomial regression analysis. The odds ratios (Exp(B)) express the odds that a member of the given group develops the given stage of periodontal disease. Controls who never smoked and early stage periodontal disease served as reference (as no periodontally healthy individuals were found in the patient group, healthy could not be used as reference). B: correlation coefficient; df: degrees of freedom

Periodontal status (reference: early)		B	df	Sig.	Exp(B)	95% CI for Exp(B)
moderate	RA- used to smoke	1.529	1	0.011	4.615	1.423–14.966
	RA- never smoked	1.442	1	0.003	4.231	1.623–11.030
	Control- used to smoke	0.932	1	0.090	2.538	0.866–7.442
	Control-never smoked	–	–	–	–	–
	Male sex	0.223	1	0.643	1.250	0.487–3.212
	Age	0.002	1	0.896	1.002	
severe	RA- used to smoke	2.788	1	0.000	16.250	3.917–67.412
	RA- never smoked	1.609	1	0.022	5.000	1.265–19.762
	Control- used to smoke	0.405	1	0.666	1.500	0.238–9.465
	Control-never smoked	–	–	–	–	–
	Male sex	0.839	1	0.867	2.315	0.703–7.619
	Age	0.028	1	0.176	1.029	0.972–1.033

## Discussion

A part of these findings is merely the corroboration of known facts. RA has been known as a risk factor for PD for some time [6–8], and our results demonstrate the same: the presence of RA in itself is enough to significantly increase the odds that the patient will develop a more severe stage of PD.

We also found that past smoking did not have a significant effect on any of the rheumatological indices and that there was no association between these indices and periodontal status. The lack of the effect of past smoking on the rheumatological status as expressed by these indices might be best explained by the time passed since the patient stopped smoking. While cigarette smoke must have been an extra immunological stimulus while the patient was still smoking, and it might as well have boosted the immunological memory against citrullinated proteins [42], these effects are unlikely to be reflected in indices characterizing a much narrower time window.

How come that no association was found between the specific RA indices and the periodontal status, while, as pointed out before, being in the RA group in itself significantly increased the odds of the more severe PD stages? Given that other studies describing larger populations found significant association with rheumatoid factor positivity and anti-citrullinated peptide antibody production [43, 44], we think that our sample size was probably too small to allow reliable assessment at the level of the individual indices, considering their greater variability.

The main finding of this study, however, is also the most difficult to explain. The finding that the FS RA patients had the highest and significant odds ratio for the severe stage of PD is really an unexpected one. From the results it appears that the effect is not mediated by the actual rheumatological status, the presence of RA with a longer period of cigarette smoke exposure in the past is enough. This suggests that long-term cigarette smoking might permanently sensitize the periodontium, but at this point we could only speculate about the possible mechanisms, and it is because of that reason that we put this up for debate.

## Conclusions

While this study definitely has its limitations, we think that our quasi-accidental finding about the effect of past smoking on periodontal health in RA deserves attention. This finding implies that long-term exposure to cigarette smoke might have a permanent sensitizing effect on the human periodontal tissues, which is not reversible by quitting smoking.

## Abbrevations

ACP: Anti-citrullinated peptide antibody
aMCV: Mutated citrullinated vimentin
anti-CCP: Anti-cyclic citrullinated peptide
anti-TNF: Anti-tumor necrosis factor
BMI: Body mass index
BOP: Bleeding on probing
CAL: Clinical attachment level
CNS: Control, never smoked
CS: Control, used to smoke
DAS28-ESR: Disease activity score
FS: Former smokers
NS: Non-smokers
OHI-S: Simplified Oral Health Index
PD: Periodontal disease
PNS: Patient, never smoked
PPAD: Peptidyl-arginine deiminase
PRD: Probing depth
PS: Patient, used to smoke
RA: Rheumatoid arthritis
RF: Rheumatoid factor
TLR-4: Toll-like receptor 4
